# P-664. Comparison of Ceftriaxone vs. Ampicillin Sulbactam for the Treatment of Community Acquired Pneumonia

**DOI:** 10.1093/ofid/ofaf695.877

**Published:** 2026-01-11

**Authors:** Maria Santana-Garces, Saher Siddiqui, Jamie G Joseph, Anqi Wang, Marcus Zervos

**Affiliations:** Henry Ford Health, Livonia, MI; Henry Ford Health, Livonia, MI; Henry Ford Health/MSU, Royal Oak, Michigan; Henry Ford Health, Livonia, MI; Henry Ford Hospital, Detroit, Michigan

## Abstract

**Background:**

Community-acquired pneumonia (CAP) is a leading cause of hospitalization and mortality in the United States, accounting for over 4.5 million healthcare visits annually. Ceftriaxone and ampicillin-sulbactam are commonly recommended β-lactams for hospitalized patients with non-severe CAP. However, direct comparisons of their clinical effectiveness remain limited. This study aims to compare clinical outcomes in hospitalized adults with CAP treated with ceftriaxone versus ampicillin-sulbactam.

**Table 1:**
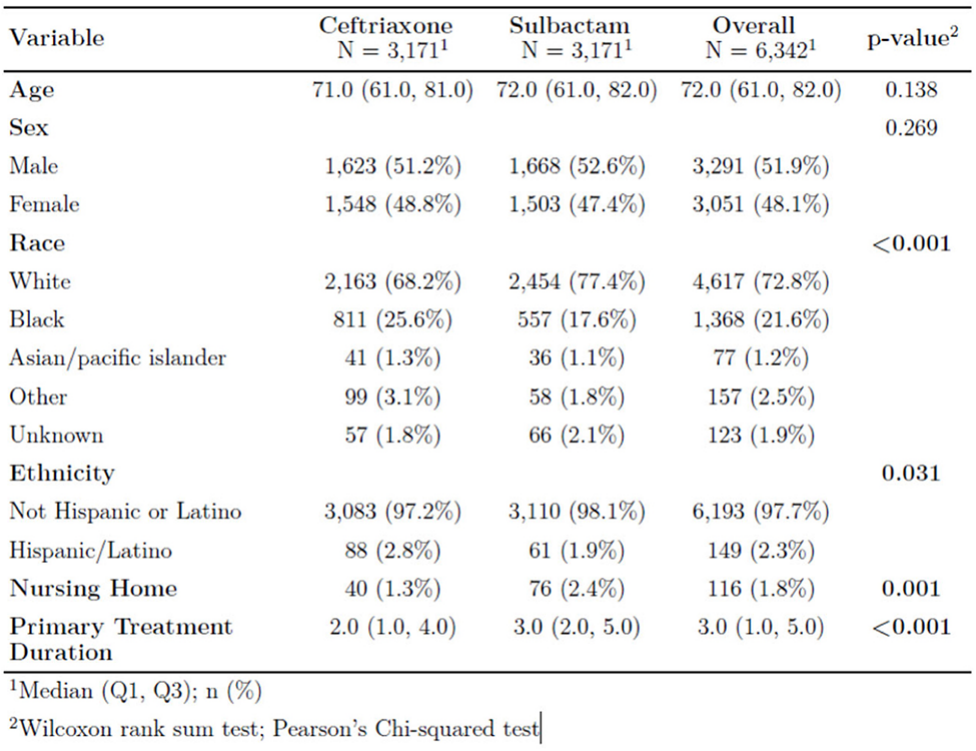
Demographics by Treatment for Matched Data

**Table 2:**
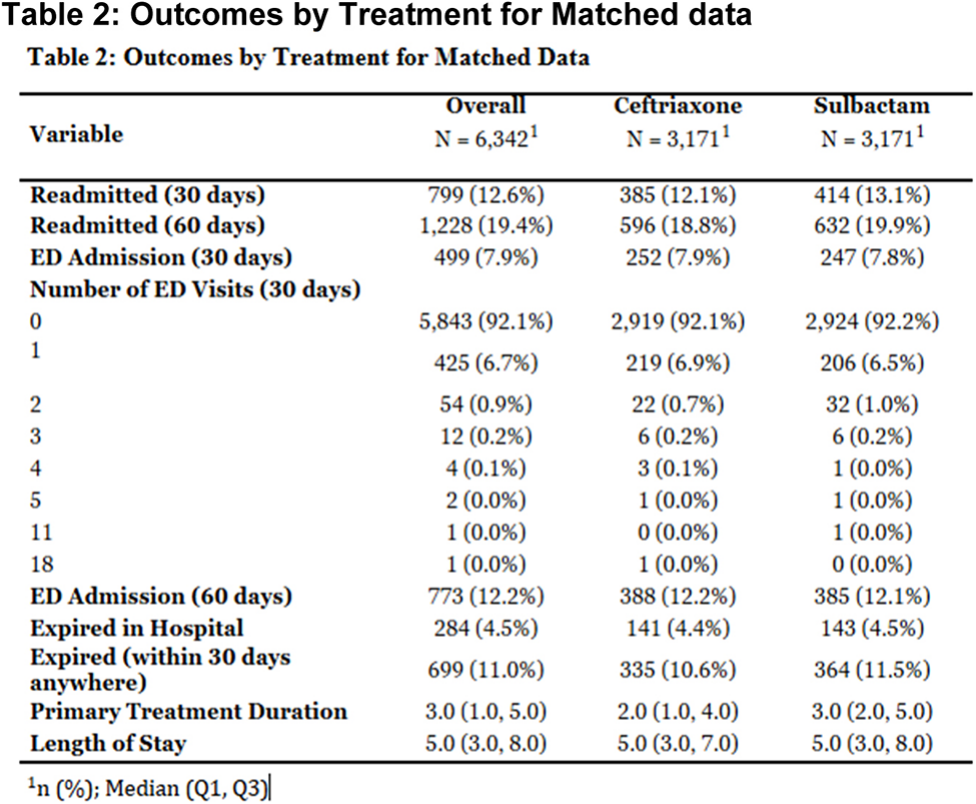
Outcomes by Treatment for Matched data

**Methods:**

This was a retrospective propensity -matched cohort study that included a total of n= 10,640 patients admitted to Henry Ford Hospital in Detroit, Michigan for treatment of CAP between January 2022 through August of 2024. The primary outcomes were in-hospital mortality and 30-day mortality. Descriptive statistics were used to summarize patient demographics, comorbidities, and outcomes for both treatment groups. Propensity score matching was used to account for confounding differences between treatment groups. A logistic regression was used to determine the association between covariates of interest and their association with in-hospital mortality or 30-day mortality.

**Results:**

A total of n=3,171 patients received ampicillin-sulbactam and n=7,469 received ceftriaxone. After propensity score matching, there was a total of n=6,342 patients with an equal number of (n= 3,171) patients in each treatment group. Overall, 4.5% of patients expired in hospital and 11% expired within 30 days. Among this cohort, 30-day readmission was 12.6% and 60-day readmission was 19.4%. Among the propensity-matched patients, there was no significant difference in 30-day mortality between those treated with ceftriaxone and ampicillin-sulbactam (OR 1.065, 95% CI 0.906–1.254, p=0.445). The adjusted OR for in hospital mortality was 1 (95% CI 0.99-1.01, p=0.9894).

**Conclusion:**

This study demonstrates no significant differences in 30-day mortality or in-hospital mortality, between patients treated with ceftriaxone and those treated with ampicillin-sulbactam. These findings support the current IDSA recommendations for therapy showing clinical equivalence of both agents as empiric treatment options for CAP.

**Disclosures:**

Marcus Zervos, MD, merck: Honoraria

